# Preferences for Long-Acting PrEP Products Among Women and Girls: A Quantitative Survey and Discrete Choice Experiment in Eswatini, Kenya, and South Africa

**DOI:** 10.1007/s10461-023-04202-0

**Published:** 2023-11-16

**Authors:** Kristen M. Little, Homaira Hanif, Sharon M. Anderson, Meredith R. Clark, Kiira Gustafson, Gustavo F. Doncel

**Affiliations:** 1 Population Services International, 1120 19th Street NW, Suite 600, Washington, DC 20036 USA; 2 Eastern Virginia Medical School, CONRAD, Norfolk, VA USA; 3 Independent Consultant, Portland, OR USA

**Keywords:** Pre-exposure prophylaxis, PrEP, Long-acting, Preferences, Discrete choice experiment

## Abstract

While oral pre-exposure prophylaxis (PrEP) can substantially reduce HIV risk, there are important barriers to uptake and adherence. We explored preferences for long-acting injectable and implantable PrEP among women and girls in Eswatini, Kenya, and South Africa. We conducted an online quantitative survey and discrete choice experiment (DCE) among adolescent girls (15–17), young women (18–29), and adult women (30–49). Participants completed a survey about their demographics and behavior and a DCE with 5 attributes (format, insertion location, number of insertions, dual-protection, and palpability). We recruited 1236 respondents (Eswatini = 420; Kenya = 350; South Africa = 493) in May 2022. Most participants were sexually active (72%), nearly 29% of whom reported recently engaging in transactional sex. 46% had heard of oral PrEP, but of those, only 16% reported having ever used it. Product format and dual-protection were significant predictors of product choice. Relative to a 2-month injection, participants had 1.76 times the odds (95% CI 1.08–2.04) of choosing a 6-month injectable, and 1.70 the odds (95% CI 1.06–1.92) of choosing a 12-month removable implant. Compared to a single-indication product, respondents had 2.46 times the odds (95% CI 1.04–2.68) of preferring a product also protecting against pregnancy, and 2.81 the odds (95% CI 1.04–3.05) of choosing a product that also protected against STIs. Adolescent girls and women in these countries showed strong preferences for longer-acting PrEP product formats, as well as those offering dual-protection. Introduction of long-acting options could improve PrEP uptake and reduce HIV burdens in east and southern African settings.

## Background

Women in east and southern Africa remain disproportionately impacted by HIV, with prevalence estimates more than twice as high as those of their male counterparts [1]. Adolescent girls (AG) and young women (YW) ages 15–24 in the region are at particular risk; for every 3 men and boys in this age group who are newly infected with HIV, there are 7 new infections among AG and YW [2]. Biomedical HIV prevention options such as oral pre-exposure prophylaxis (oral PrEP) may help to reduce new HIV infections among women and girls in these contexts if they are made accessible and can be used effectively [3]. Recognizing this potential, in 2016 the World Health Organization (WHO) expanded their original recommendations to offer PrEP to men who have sex with men (MSM) and sero-discordant couples to include “all individuals at substantial risk of HIV infection”[4], and in 2021 further expanded this recommendation to cover any individual who requests PrEP [5].

While oral PrEP can reduce a user’s risk of acquiring HIV by more than 90%, effectiveness varies considerably across populations, with several studies finding low or no effectiveness and low rates of PrEP adherence among women in sub-Saharan Africa [6–9]. Oral PrEP effectiveness is highly dependent on adherence levels, and models predict women, unlike men, need to take 6–7 pills per week in order to achieve high protective levels [10]. Unique adherence barriers among women and girls have likely contributed to demonstrably lower PrEP effectiveness estimates for heterosexual women in controlled trials [11]. In addition to supply-side barriers, including policies limiting PrEP access to narrowly defined groups, subsequent research has identified numerous demand-side barriers to oral PrEP uptake and continuation among women and girls. These include stigma related to sexual activity (especially among adolescent girls) [12, 13], assumptions around one’s HIV status related to taking antiretrovirals [14], pill burden [15, 16], the need for regular clinic visits [17, 18], low perceived risk of HIV [19, 20], and service delivery largely centralized in HIV, STI, or key-population-focused clinic spaces and programs where girls and women don’t often seek care [21].

New or emerging biomedical HIV prevention technologies, including vaginal rings [22], long-acting injectables [23], and implants [24], may help to address some of these adherence barriers by providing women with options that are not user-mediated and provide protection over a longer period. While they are still early in the research and development (R&D) phase, PrEP implants will offer women a particularly long-acting option, providing protection against HIV acquisition for months or even years at a time [25]. HIV PrEP implants are designed to be implanted in the body much like a contraceptive implant or long-acting injectables. They may even be formulated with a contraceptive hormone, which would provide users with added protection against unplanned pregnancies [25]. Previous research among adolescent girls, young women, and female sex workers in South Africa found the concept of a PrEP implant to be highly acceptable. Target end-users expressed strong preferences for an implant product with a high level of effectiveness, as well as a product that produced only mild side-effects [26].

Building on this work, additional research was needed to further expand the geographic scope of previous preferences studies, and to better understand target end-user preferences for additional product characteristics of interest, such as format (e.g., injection vs. implant; dissolvability vs. removability) and dual vs. single indication (e.g., HIV only, or HIV protection plus protection against STIs or pregnancy) likely to drive product uptake.

To inform ongoing R&D efforts, we conducted an online quantitative survey and discrete choice experiment (DCE) with adolescent girls (AG), young women (YW), and adult women (AW) in 3 countries in east and southern Africa with a high burden of HIV: Eswatini, Kenya, and South Africa. Specifically, we sought to determine women’s and girls’ preferences for implantable PrEP products, and to determine if those preferences vary by age, geography, or prior use of long-acting contraceptive implants or oral PrEP. Understanding women’s and girls’ values and preferences around long-acting HIV prevention products will be critical to developing options that meet their needs and effectively address barriers to adoption. Given high HIV incidence rates among young women, designing products that consider their preferences is critical to reducing the burden of HIV and empowering women and girls by giving them more control over their own health.

## Methods

### Study Setting and Population

We recruited participants through online market research databases in each country. Database members tend to be more likely to reside in an urban area, have higher educational attainment, and belong to higher wealth quintiles relative to the general population. Because of this, we limited recruitment to a single major population center in each country: Durban, Kwazulu Natal (South Africa), Mbabane (Eswatini) and Nairobi (Kenya). These urban centers were purposively selected to include geographies with large populations, relatively high HIV burdens, ongoing oral PrEP interventions, and where the target populations were relatively more familiar with oral PrEP [32]. Adult respondents were randomly selected from the database and were eligible for the study if they were female, ages 18–29 (YW) or 30–49 (AW), were members of the market research database (and provided confirmation of their identity), were current residents of the study country/urban center, had access to an internet enabled device, and were willing/able to provide electronic informed consent.

Because individuals < 18 were not included in the market research databases, AG were recruited to the study via their parents/guardians. Randomly selected adult members of the databases from the study areas (not previously selected as survey respondents themselves) were screened to see if they had a potentially eligible adolescent girl in their household. Interested parents/guardians were asked to provide parental informed consent for their daughter to participate, and AG provided informed assent to participate. AG were eligible if they were female, ages 15–17, had a parent/guardian in the market research database, were current residents of a study country/urban center, had access to an internet enabled device, and were able/willing to provide informed assent and had parental consent to participate.

Based on DCE sample size convention, we estimated we would need approximately 200 respondents per population segment for this study [27]. Our sample was divided evenly across the three study groups (AG, YW, and AW) and 3 country settings.

### Quantitative Survey and Discrete Choice Experiment

The quantitative survey collected data on participants’ socio-demographic characteristics (marital status, education, income, household SES), awareness and use of analogous products (e.g., the contraceptive implant and oral PrEP), contraceptive use, perceived HIV risk and HIV testing history, and current HIV prevention approaches, if any.

**Fig. 1:**
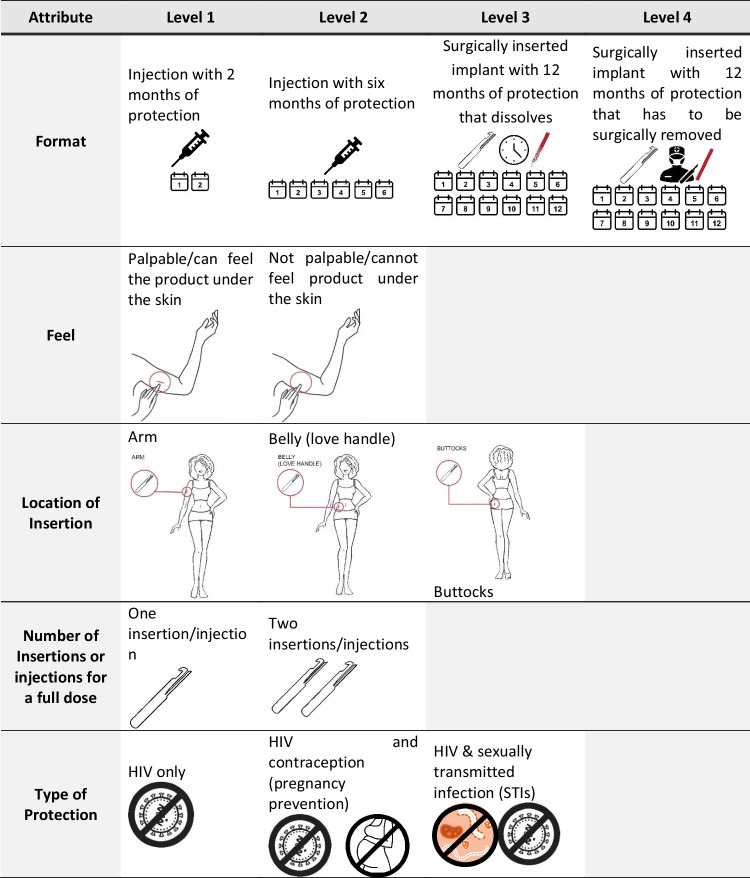
PrEP implant product attributes and levels included in the discrete choice experiment

The DCE design was informed by our previous research on PrEP implants in South Africa [26, 28], a literature review, stakeholder engagement, and an assessment of current product development information needs to refine the list of product characteristics to be tested. Selection of the final list of attributes and levels attempted to find a balance between maximizing the data gathered by the survey with the degree of cognitive burden on participants. The experiment design was developed using Ngene software. We used a D-efficient design approach for analysis of main effects, while minimizing overlap and dependence. The resulting design contained 5 attributes, with two to four possible levels each (Fig. 1). To further limit cognitive fatigue, each participant was randomized to one of 3 blocks of 12 choice sets during the survey. Survey instruments and DCE images were pilot tested with the target population prior to fielding.

### Study Design and Statistical Analysis

Participants completed the quantitative survey and DCE online via an electronic survey software platform. A trained study staff member was available via phone if the respondent encountered any challenges or had questions. During the DCE, participants were presented choice tasks with illustrative graphics. For each choice task, participants selected their preference between the two unlabeled product options and then indicated if they would rather use the option selected or their current HIV prevention approach (if any). In addition to their block of 12 choice tasks, all respondents completed an additional choice task designed to check comprehension and attention. In this 13^th^ choice task, all of the hypothesized most-preferred levels were used for one product’s attributes, and the least-preferred for the other.

Quantitative survey data were analyzed descriptively using means and frequencies and appropriate tests of association (e.g., t-tests and chi-square tests). Household wealth quintile was estimated using the EquityTool [29]. Stated preference data were analyzed using fixed effects logistic regression in Stata 15.0 (College Station, TX). Stratification analyses were conducted to determine whether differences in stated preferences existed between end-user groups. Differences in stated preference estimates by strata were evaluated using Chow and Wald tests.

### Ethics

This protocol was approved by the relevant ethical review boards in the selected countries, including the Population Services International Research Ethics Board (PSI REB), the Eswatini Health and Human Research Review Board, the Amref Institutional Review Board in Kenya, and the University of the Witwatersrand HREC (Medical) in South Africa. All respondents provided electronic or verbal informed consent to participate (AG < 18 provided informed assent and had parental consent to participate).

## Results

Between May 13—May 31, 2022, we recruited a total of 1263 respondents (Table 1) across Eswatini (n = 420, 33.3%), Kenya (n = 350, 27.7%), and South Africa (n = 493, 39.0%). We enrolled a total of 348 (27.6%) adolescent girls (AG), 515 (40.8%) young women (YW), and 400 (31.7%) adult women (AW). Most respondents belonged to urban wealth quintiles 3 (18.8%) or 4 (57.2%) and had obtained their metric (20.8%) or completed university or graduate school (38.9%). Most respondents reported being single (43.8%) or having a regular (non-spouse) partner (22.2%), and 72% reported ever having been sexually active. Among those reporting sexual activity, over one-quarter (227/792, 28.7%) reported engaging in transactional sex in the previous year, a proportion that was significantly higher in Kenya (34.2%) and Eswatini (31.4%) than in South Africa (21.9%, p = 0.006). AG were significantly more likely than young and adult women to report a recent history of transactional sex (44.9% vs. 26.6% or 23.4%, respectively, p < 0.001).

**Table 1:** Participant demographic characteristics, by country

Characteristic	Total (N = 1263) N (%)	eSwatini (n = 420, 33%) n(%)	Kenya (n = 350, 28%) n (%)	South Africa (n = 493, 39%) n (%)	p-value
Age group (N = 1263)					
Adolescent girls (15–17)	348 (27.6)	104 (24.8)	144 (41.1)	100 (20.3)	< 0.001
Young women (18–29)	515 (40.8)	214 (41.6)	104 (29.7)	197 (40.0)	
Adult women (30–49)	400 (31.7)	102 (24.3)	102 (29.1)	196 (39.8)	
HH urban wealth quintile* (N = 1263)					
1	43 (3.4)	35 (8.3)	5 (1.4)	3 (0.6)	< 0.001
2	86 (6.8)	50 (11.9)	18 (5.1)	18 (3.7)	
3	237 (18.8)	77 (18.3)	71 (20.3)	89 (18.1)	
4	723 (57.2)	216 (51.4)	124 (35.4)	383 (77.7)	
5	174 (13.8)	42 (10.0)	132 (37.7)	0 (0.0)	
Highest level of education completed (N = 1263)					
Primary	193 (15.3)	32 (7.6)	100 (28.6)	61 (12.4)	< 0.001
Secondary	316 (25.0)	145 (34.5)	68 (19.4)	103 (20.9)	
Metric	263 (20.8)	121 (28.8)	16 (4.6)	126 (25.6)	
University	376 (29.8)	75 (17.9)	137 (39.1)	164 (33.3)	
Graduate School	115 (9.1)	47 (11.2)	29 (8.3)	39 (7.9)	
Marital status (N = 1248)					
Husband/spouse	204 (16.4)	58 (13.9)	71 (20.5)	75 (15.5)	< 0.001
Regular partner	277 (22.2)	99 (23.7)	56 (16.2)	122 (25.2)	
Casual partner	220 (17.6)	75 (17.9)	44 (12.7)	101 (20.9)	
Single	547 (43.8)	186 (44.5)	175 (50.6)	186 (38.4)	
Ever had sexual intercourse (N = 1245)	896 (72.0)	345 (82.7)	231 (66.8)	320 (66.4)	< 0.001
Number of partners in last 12 months, Mean (SD) (N = 834)	2.13 (5.76)	3.21 (8.51)	1.27 (0.96)	1.57 (3.41)	0.0001
Engaged in transactional sex in last 12 months** (N = 792)	227 (28.7)	100 (31.4)	65 (34.2)	62 (21.9)	0.006

*Urban Wealth Quintiles calculated separately for each country using the EquityTool (equitytool.org)

**Transactional sex question asked respondents “In the past 12 months have you entered into a sexual relationship with a man mainly in order to get things that you needed, money, gifts, or other things that are important to you?”

### Modern Contraceptives & Experience with Implants and Injectables

Self-reported family planning (FP) use among sexually active participants was > 80%, ranging from 74% in South Africa to 90% in Kenya (p < 0.001). A history of having ever used a method to delay/prevent pregnancy was similar across age groups (p = 0.425, Table 2). Among current FP users, 9% of women reported using the contraceptive implant, ranging from 6% in South Africa to 11% in Eswatini (p = 0.277), though 17% (209/1229) of the sample reported ever having used an implant. AW were significantly more likely to have implant experience (26.1%) compared to YW (16.6%) or AG (7.3%, p < 0.001). Women and girls frequently reported a history of injectable contraceptive use, ranging from 23% in Kenya to 39% in South Africa (p < 0.001). As with implants, ever using injectables was most frequently reported by AW (45.5%) compared to their younger counterparts (YW: 31.7%; AG: 16.1%; p < 0.001).

**Table 2:** Modern contraceptives and experience with contraceptive implants and injectables by age group

Characteristic	Total (N = 1263) N (%)	AG (n = 348, 28%) n (%)	YW (n = 515, 41%) n (%)	AW (n = 400, 32%) n (%)	p-value
Used a condom at last sex* (N = 883)	465 (52.7)	118 (71.1)	223 (56.5)	124 (38.5)	< 0.001
Ever used a family planning method* (N = 854)	694 (81.3)	132 (83.0)	318 (82.4)	244 (79.0)	0.425
Contraceptive implant	80 (11.5)	6 (4.6)	33 (10.4)	41 (16.8)	0.001
Injectable contraceptive	124 (17.9)	18 (13.6)	58 (18.2)	48 (19.7)	0.336
Oral contraceptive pill	210 (30.3)	27 (20.5)	90 (28.3)	93 (38.1)	0.001
Male condoms	453 (65.3)	97 (73.5)	221 (69.5)	135 (55.3)	< 0.001
Female condoms	61 (8.8)	7 (5.3)	35 (11.0)	19 (7.8)	0.119
Emergency contraceptive pills	197 (28.4)	35 (26.5)	98 (30.8)	64 (26.2)	0.425
Intrauterine device (IUD)	42 (6.1)	1 (0.8)	16 (5.0)	25 (10.3)	0.001
Withdrawal	230 (33.1)	26 (19.7)	125 (39.3)	79 (32.4)	< 0.001
Other method	5 (0.7)	0 (0)	3 (0.9)	2 (0.8)	0.545
Currently using a family planning method* (N = 694)	562 (81.0)	111 (84.1)	265 (83.3)	186 (76.2)	0.062
Contraceptive implant	49 (8.7)	5 (4.5)	21 (7.9)	23 (12.4)	0.055
Injectable contraceptive	91 (16.2)	19 (17.1)	40 (15.1)	32 (17.2)	0.800
Oral contraceptive pill	135 (24.0)	19 (17.1)	72 (27.2)	44 (23.7)	0.113
Male condoms	320 (56.9)	83 (74.8)	160 (60.4)	77 (41.4)	< 0.001
Female condoms	37 (6.6)	3 (2.7)	23 (8.7)	11 (5.9)	0.093
Emergency contraceptive pills	98 (17.4)	24 (21.6)	53 (20.0)	21 (11.3)	0.024
IUD	26 (4.6)	1 (0.9)	7 (2.6)	18 (9.7)	< 0.001
Withdrawal	131 (23.3)	21 (18.9)	79 (29.8)	31 (16.7)	0.002
Other method	13 (2.3)	1 (0.9)	4 (1.5)	8 (4.3)	0.082
Ever heard of the contraceptive implant	733 (58.3)	143 (41.1)	336 (65.6)	254 (63.8)	< 0.001
Ever used the contraceptive implant (N = 1229)	209 (17.0)	25 (7.3)	83 (16.6)	101 (26.1)	< 0.001

*Among sexually active participants

### HIV Risk, Prevention, and PrEP Experience

Most respondents (74%) had previously been tested for HIV, ranging from 65% in South Africa to 87% in Eswatini (p < 0.001, Table 3). AW (80.7%) and YW (80.0%) were significantly more likely than AG (57.5%) to report having ever tested for HIV (p < 0.001). Most respondents felt it would be “serious” (18.4%) or “very serious” (50.2%) to acquire HIV, ranging from 55% in Eswatini to 87% in Kenya (p < 0.001). AG were significantly more likely to rate HIV acquisition as “serious” or “very serious” (77.1%) relative to YW (61.9%) or AW (70.1%, p < 0.001). Male condoms were the most common strategy to prevent HIV, reported by 50% of respondents, followed by abstinence (29.5%) and limiting their number of sexual partners (24.4%).

**Table 3:** HIV testing and risk perception by country

Characteristic	Total (N = 1263) N (%)	eSwatini (n = 420, 33%) n (%)	Kenya (n = 350, 28%) n (%)	South Africa (n = 493, 39%) n (%)	p-value
Ever tested for HIV (N = 1254)	928 (74.0)	361 (86.6)	250 (71.8)	317 (64.8)	< 0.001
Time since most recent HIV test (N = 928)					< 0.001
< 6 months	374 (40.9)	139 (39.0)	76 (30.5)	159 (51.3)	
6–12 months	228 (24.9)	110 (30.9)	48 (19.3)	70 (22.6)	
> 1–2 years	167 (18.3)	82 (23.0)	53 (21.3)	32 (10.3)	
> 2–5 years	85 (9.3)	20 (5.6)	41 (16.5)	24 (7.7)	
> 5 years	61 (6.7)	5 (1.4)	31 (12.5)	25 (8.1)	
Confidence in ability to remain HIV negative* Mean (SD)	1.9 (1.2)	2.1 (1.2)	1.6 (1.1)	2.0 (1.3)	< 0.001
How serious would it be to get HIV					< 0.001
Not at all serious	123 (10.4)	63 (16.0)	15 (4.6)	45 (9.7)	
Somewhat serious	155 (13.1)	73 (18.6)	17 (5.2)	65 (14.1)	
Neither serious nor unserious	94 (7.9)	42 (10.7)	10 (3.0)	42 (9.1)	
Serious	218 (18.4)	60 (15.3)	58 (17.6)	100 (21.7)	
Very serious	595 (50.2)	155 (39.4)	230 (69.7)	210 (45.5)	
Perceived risk of ever contracting HIV** Mean (SD)	2.7 (2.3)	2.9 (2.3)	2.6 (2.4)	2.7 (2.2)	0.0981
What actions (if any) being taken to prevent HIV					
Abstinence	373 (29.5)	110 (26.2)	163 (46.6)	100 (20.3)	< 0.001
Reducing sexual partners	308 (24.4)	109 (26.0)	91 (26.0)	108 (21.9)	0.260
Having partners close to one’s age	94 (7.4)	31 (7.4)	9 (2.6)	54 (11.0)	< 0.001
Male condoms	630 (49.9)	246 (58.6)	167 (47.7)	217 (44.0)	< 0.001
Female condoms	207 (16.4)	59 (14.1)	49 (14.0)	99 (20.1)	0.018
Partner testing	274 (21.7)	62 (14.8)	81 (23.1)	131 (26.6)	< 0.001
Partner on treatment/virally suppressed	33 (2.6)	8 (1.9)	2 (0.6)	23 (4.7)	0.001
Oral PrEP	66 (5.2)	30 (7.1)	6 (1.7)	30 (6.1)	0.002
Avoiding needle sharing	330 (26.1)	127 (30.2)	87 (24.9)	116 (23.5)	0.058
Avoiding multiple, concurrent partners	279 (22.1)	82 (19.5)	83 (23.7)	114 (23.1)	0.294
Vaginal gels/rings	33 (2.6)	7 (1.7)	8 (2.3)	18 (3.7)	0.156
Other	54 (4.3)	18 (4.3)	14 (4.0)	22 (4.5)	0.948

*Scale from 1 to 5, with 1 being “I will not have HIV by the end of my lifetime” and 5 being “I will have HIV by the end of my lifetime”

**Scale from 1 to 10, with 1 being “No risk” and 10 being “Very high risk”

Nearly half (45.5%) of the respondents had previously heard of oral PrEP. This proportion ranged from 37% in South Africa to 57% in Eswatini (p < 0.001). YW (53.4%) and AW (48.5%) were significantly more likely than AG (30.5%) to have heard of oral PrEP (p < 0.001). Roughly 16% (90/565) of those familiar with oral PrEP (7.1% of total sample) reported either current or previous oral PrEP use, ranging from 10% in Kenya to 19% in South Africa (p = 0.082). Among those who had never used oral PrEP, the most common reasons reported for non-use were a lack of awareness (38.6%) and not perceiving oneself as at risk for HIV (31.3%). However, roughly half of non-users indicated that they would be “likely” (20.3%) or “very likely” (28.7%) to use oral PrEP in the future. “Likely” or “very likely” future oral PrEP use was most commonly reported among AG (58.1%) vs. YW (44.6%) or AW (44.6%, p = 0.008).

### Preferences for a PrEP Implant Product

When asked about their likelihood of using a PrEP implant product were one available in the future, more than half (59.8%) of respondents said they would “Very likely” (26.2%) or “Somewhat likely” (33.6%) to use it (Table 4). As with oral PrEP, the proportion of “likely” or “very likely” potential users was higher among AG (66.6%) compared to YW (58.0%) or AW (56.3%, p = 0.032). Most respondents (60.1%) reported that they would prefer to get a PrEP implant from a family planning (FP) provider vs. an HIV care provider (35.0%) or another type of provider (4.9%). Preferences for FP providers did not differ significantly by age group but ranged from 48% in Kenya to 72% in Eswatini (p < 0.001).

**Table 4:** Oral pre-exposure prophylaxis awareness, use, and preferences

Characteristic	Total (N = 1263) N (%)	eSwatini (n = 420, 33%) n (%)	Kenya (n = 350, 28%) n (%)	South Africa (n = 493, 39%) n (%)	p-value
Heard of oral PrEP (N = 1244)	566 (45.5)	235 (56.8)	153 (44.0)	178 (36.9)	< 0.001
Heard of other PrEP products	566 (44.8)	235 (56.0)	153 (43.7)	178 (36.1)	< 0.001
Vaginal rings	129 (10.2)	35 (8.3)	52 (14.9)	42 (8.5)	0.003
Injectable PrEP	169 (13.4)	65 (15.5)	49 (14.0)	55 (11.2)	0.149
PrEP implant	231 (18.3)	120 (28.6)	40 (11.4)	71 (14.4)	< 0.001
Other PrEP product	196 (15.5)	92 (21.9)	52 (14.9)	52 (10.6)	< 0.001
Ever used oral PrEP (N = 565)	90 (15.9)	40 (17.1)	16 (10.5)	34 (19.1)	0.082
Why haven’t you used oral PrEP (N = 1173)					
Unaware of it	453 (38.6)	144 (37.9)	156 (46.7)	153 (33.3)	0.001
Didn’t know where to get it	106 (9.0)	29 (7.6)	30 (9.0)	47 (10.2)	0.423
Not at risk for HIV	367 (31.3)	102 (26.8)	114 (34.1)	151 (32.9)	0.071
Partner wouldn’t want me to	58 (4.9)	10 (2.6)	9 (2.7)	39 (8.5)	< 0.001
I don’t like taking pills	135 (11.5)	57 (15.0)	18 (5.4)	60 (13.1)	< 0.001
I don’t want to test for HIV	63 (5.4)	12 (3.2)	11 (3.3)	40 (8.7)	< 0.001
It’s too expensive	56 (4.8)	3 (0.8)	9 (2.7)	44 (9.6)	< 0.001
It’s too inconvenient	58 (4.9)	22 (5.8)	5 (1.5)	31 (6.8)	0.002
Concerned about side effects	147 (12.5)	62 (16.3)	28 (8.4)	57 (12.4)	0.006
Concerned about effectiveness	53 (4.5)	25 (6.6)	17 (5.1)	11 (2.4)	0.012
Other	83 (7.1)	33 (8.7)	34 (10.2)	16 (3.5)	< 0.001
Don’t know	77 (6.6)	35 (9.2)	13 (3.9)	29 (6.3)	0.016
How likely would you be to take oral PrEP in the future* (N = 1104)					< 0.001
Very likely	224 (20.3)	75 (20.1)	57 (18.2)	92 (22.1)	
Likely	317 (28.7)	118 (31.6)	85 (27.2)	114 (27.3)	
Neither likely nor unlikely	246 (22.3)	106 (28.3)	52 (16.6)	88 (21.1)	
Unlikely	184 (16.7)	55 (14.7)	66 (21.1)	63 (15.1)	
Very unlikely	133 (12.1)	20 (5.4)	53 (16.9)	60 (14.4)	

*Among never-users

**Fig. 2:**
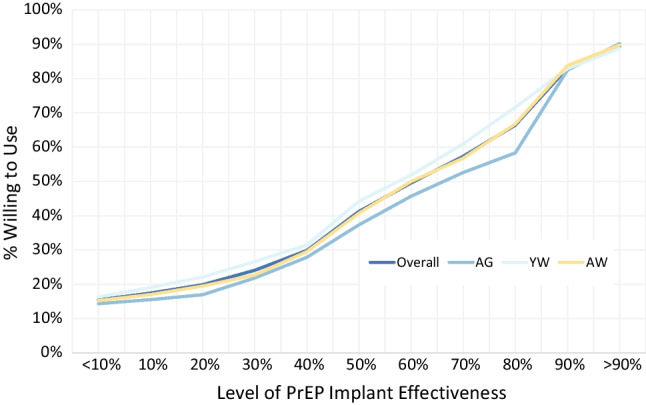
Stated willingness to use a PrEP implant at each level of effectiveness, by age group

Stated willingness to use a PrEP implant was strongly positively correlated with implant effectiveness (Fig. 2). For every 10% increase in product effectiveness, the proportion of respondents willing to use it increased by approximately 8%. When asked about the importance of a range of implant product attributes, product effectiveness was among the features most frequently rated as “very important” (70.9%), along with duration of protection (70.5%), and removability/dissolvability (75%).

When asked about their preferences for a dissolvable implant product (that couldn’t be removed after insertion) versus a non-dissolvable product that would have to be surgically removed when no longer needed/wanted, respondents were split (Table 5). When asked to choose between a 12-month dissolvable implant, 12 month surgically removable implant, or a 6-month injection, the dissolvable implant was most popular (selected by 40% of respondents) followed by the 6-month injection and surgically removable implant (both 30%). The popularity of a dissolvable implant varied significantly by country, ranging from 30% in Eswatini to 45% in South Africa (p < 0.001).

**Table 5:** PrEP implant preferences

Characteristic	Total (N = 1263) N (%)	eSwatini (n = 420, 33%) n (%)	Kenya (n = 350, 28%) n (%)	South Africa (n = 493, 39%) n (%)	p-value
Likelihood of using a PrEP implant in future (N = 1240)					0.053
Very likely	325 (26.2)	112 (27.1)	75 (21.6)	138 (28.8)	
Likely	417 (33.6)	144 (34.8)	114 (32.9)	159 (33.2)	
Neither likely nor unlikely	265 (21.4)	87 (21.0)	81 (23.3)	97 (20.3)	
Unlikely	144 (11.6)	49 (11.8)	52 (15.0)	43 (9.0)	
Very unlikely	89 (7.2)	22 (5.3)	25 (7.2)	42 (8.8)	
Where would you rather get the PrEP implant from					< 0.001
FP provider/clinic	759 (60.1)	304 (72.4)	167 (47.7)	288 (58.4)	
HIV provider/clinic	442 (35.0)	90 (21.4)	172 (49.1)	180 (36.5)	< 0.001
Other	62 (4.9)	26 (6.2)	11 (3.1)	25 (5.1)	
Implant format preference					
Dissolvable implant, not removable, 12 months protection	502 (39.8)	126 (30.0)	155 (44.3)	221 (44.8)	
Non-dissolving implant, surgical removal, 12 months protection	382 (30.3)	164 (39.1)	83 (23.7)	135 (27.4)	
Injection, 6 months protection	379 (30.0)	130 (31.0)	112 (32.0)	137 (27.8)	

*p < *0.05*

**p < *0.01*

***p < *0.001*

**Fig. 3:**
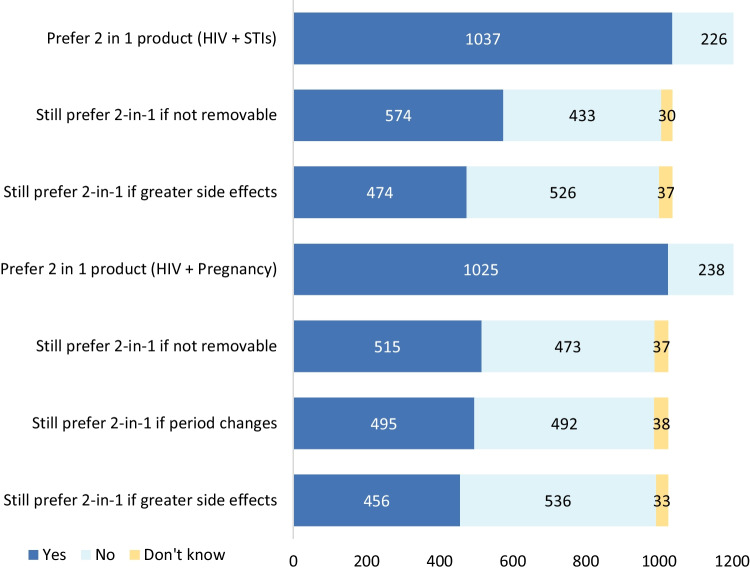
Preferences for dual-protection vs. single indication products

Respondents expressed clearer preferences for dual-protection products (Fig. 3). More than 80% of respondents would prefer a product that protected against HIV and pregnancy (relative to one that would only protect against HIV), a preference that did not vary significantly by age group (p = 0.833), though was slightly more popular in South Africa (84.8%) relative to Kenya (79.4%) or Eswatini (78.3%, p = 0.028). More than 80% of respondents would also prefer a dual-protection product that prevented HIV and other sexually transmitted infections (STIs). This did not vary significantly by age or country. Preference for both dual-protection products diminished somewhat if they would cause greater side-effects than using multiple single indication products simultaneously, falling from around 80% to 45% for both dual-protection options. A similar pattern was observed if the dual-protection product was dissolvable, and could not be removed (e.g., in the event the user wanted to get pregnant or was no longer at risk for STIs).

**Fig. 4:**
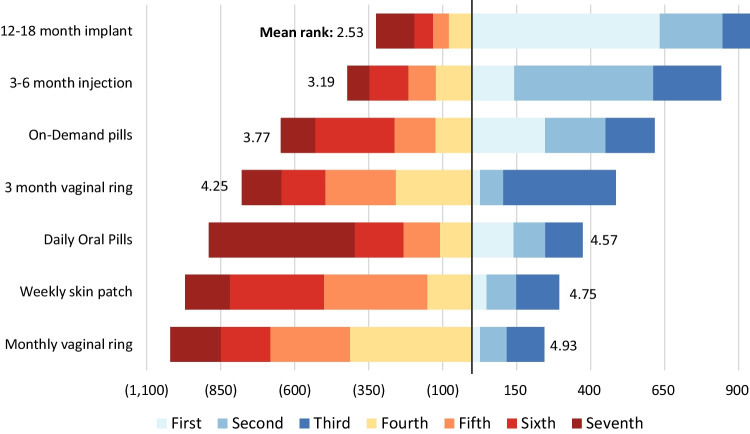
Ranking of potential PrEP product formats

When ranking their preferences for PrEP products, including implants, long-acting injectables, on-demand and daily oral pills, vaginal rings, and patches (Fig. 4), participants most preferred a PrEP implant with 12–18 months of protection (average rank: 2.53), followed by an injectable lasting 3–6 months (3.19), on-demand oral pills (3.77), and a vaginal ring with 3 months of protection (average rank: 4.25). Daily oral pills (average rank: 4.93), weekly skin patches (4.75), and a monthly vaginal ring (4.57) were ranked lowest, on average. While preferences were similar across age groups on average, AW tended to rank long acting injectables higher than other groups (3.06 vs. 3.39 for AG and 3.17 for YW, p = 0.0338).

### Discrete Choice Experiment

**Fig. 5:**
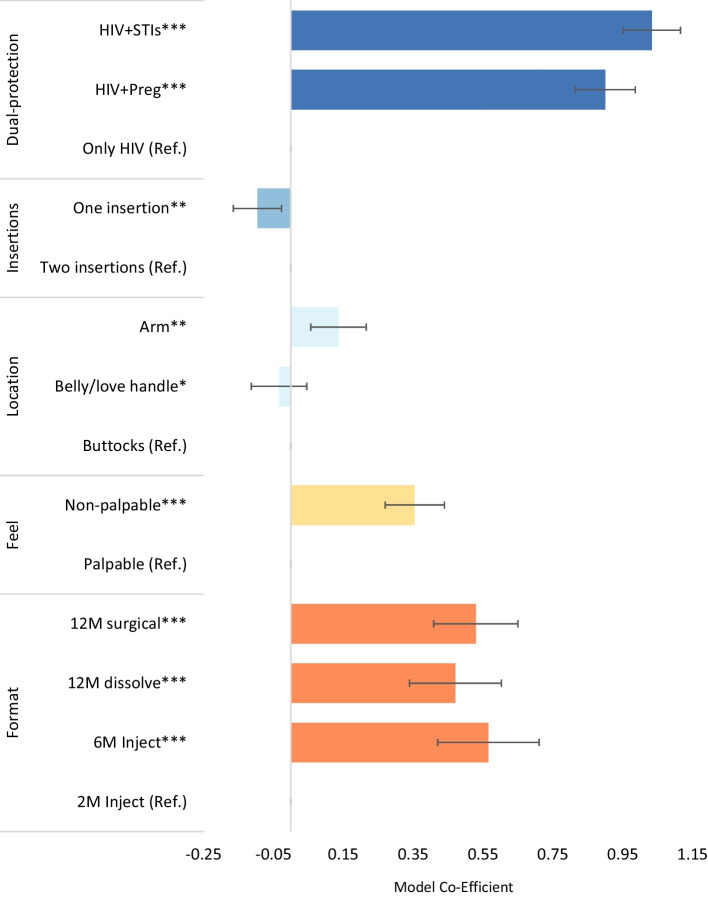
DCE results, overall study population

On the DCE respondents’ preferences were significant influenced by dual-protection, product palpability, and product format (Fig. 5). Respondents had 2.5 (95% CI 2.26–2.68) times the odds of selecting a product that protected against HIV and pregnancy, and 2.8 (95% CI 2.59–3.05) times the odds of selecting a product protecting against HIV and other STIs, relative to a single indication product, adjusting for other product features. Respondents had 1.4 (95% CI: 1.31–1.55) times the odds of choosing a product that was not palpable beneath the skin compared to one that could be felt. Participants also expressed preferences for products with longer durations of protection. Relative to an injectable with two months of protection, respondents had 1.7 (95% CI 1.51–1.92) times the odds of selecting a 12 month surgically removable implant, 1.6 (95% CI 1.40–1.83) times the odds of choosing a 12-month dissolvable product, and 1.8 (95% CI 1.52–2.04) times the odds of selecting a 6-month injectable product.

**Fig. 6:**
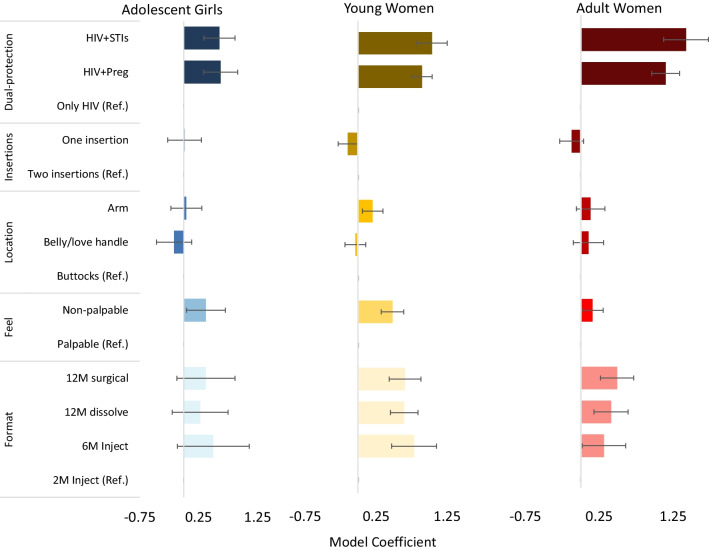
Discrete choice experiment results, by age group

Preferences were broadly similar across age groups with a few exceptions (Fig. 6). While YW and AW expressed significant preferences for long-acting product formats (relative to a two-month injectable product), format was not a significant predictor of product preferences for AG. All age groups expressed preferences for dual-protection products relative to a product protecting only against HIV, though these preferences were stronger among YW and AW. Compared to a single indication product, YW had 2.8 (95% CI 2.28–3.49) times greater odds of choosing a product protecting against HIV and STIs, and AW had 4.2 times greater odds (95% CI 3.08–5.65), compared to 1.8 (95% CI 1.40–2.38) for AG. Palpability was also a stronger predictor of product preference for AG (UR: 1.45, 95% CI 1.04–2.02) and YW (UR: 1.62, 95% CI 1.38–1.90), compared to AW (UR: 1.17, 95% CI 1.02–1.36). Location of insertion and number of insertions were not significant predictors of product preferences for AG, YW, or AW.

## Discussion

While nearly half of the respondents in our study were aware of oral PrEP, less than 10% of the study sample had ever used it. This reflects the relatively slow roll-out of PrEP in sub-Saharan African settings, driven by under-prepared health systems, regulatory hurdles, limited community engagement, and insufficient funding [30, 31]. PrEP use among heterosexual girls and women may have been further slowed by low risk perception [32, 33] and the narrow targeting of oral PrEP to specific high-risk groups (e.g., female sex workers, sero-discordant couples) when it was originally introduced. In 2015, the World Health Organization (WHO) recommended PrEP for populations at ‘substantial risk’ of HIV infection (HIV incidence greater than 3 per 100 person–years in the absence of PrEP) [4]. In 2021, WHO broadened PrEP recommendations beyond such narrowly defined groups, which enables more equitable access and is likely to be less stigmatizing than targeting specific risk groups [5]. These and other policy changes [34] along with investments in further oral PrEP scale-up have resulted in nearly three-quarters of a million cumulative people initiating PrEP across Eswatini, Kenya, and South Africa to date [35].

Of those with oral PrEP experience in our study, nearly half were no longer taking it. While many users reported stopping due to a reduction in their HIV risk, cessation was frequently due to side-effects, dislike of daily pill-taking, and inconvenience. Among those without PrEP experience, in addition to a lack of awareness and low HIV risk perception, the most common reasons for not having used oral PrEP included disliking taking pills and concerns about product side effects. These barriers to PrEP uptake and continuation have been commonly reported in the literature and appear especially salient for adolescent girls and young women [21, 32, 36, 37].

In addition to demand generation and AGYW-friendly service delivery options, these barriers highlight the need for PrEP product options that better meet the needs and preferences of women and girls across the life course. Our DCE found that dual-protection, product palpability, and product format significantly predicted preferences for PrEP products across age groups and countries. Importantly, women and girls showed strong preferences for products that had a high effectiveness and a longer duration of protection. Other DCEs, including those conducted among youth and adolescents, have also observed preferences for longer-acting PrEP products and those requiring less frequent dosing [38, 39]. Recent evidence among cis-gender women supports that longer-acting products more effectively prevent HIV acquisition, due in part to improved adherence relative to a daily pill [40, 41]. While our study found that all longer-acting formats (6-month injectable, 12-month biodegradable implant, 12-month surgically removable implant) were preferred over a 2-month injectable, we did not see a significant difference in preferences between the three longer-acting product formats, and our survey findings suggest substantial heterogeneity in format preferences. Offering women and girls the choice of products among an assortment of PrEP formats, including oral pills, vaginal rings and gels, and long-acting injectable or implantable options may improve uptake and continuation and reduce HIV incidence in this population. Increased choice has been associated with increased contraceptive use [42], and among AGYW who use them long-acting reversible contraceptives have higher efficacy, continuation rates, and satisfaction (relative to short-acting methods) [43].

Women and girls in our study strongly preferred products that protected against HIV as well as pregnancy or other STIs, compared to products only protecting against HIV. Development of multi-purpose prevention technologies (MPTs) has accelerated in recent years, with several products in the development pipeline including both long-acting and on-demand options [44, 45]. MPTs have the potential to both better appeal to target end-users (46–48) and to be cost-effective [49] relative to single-indication products. A recent costing study found that MPTs, including long-acting injectable antiretrovirals (ARVs), would likely be cost-effective among female sex workers and young women ages 16–24 in South Africa [50]. Another modeling study from South Africa found that provision of long-acting PrEP delivery to all HIV-negative injectable contraceptive users would be cost-effective at low drug prices, or when targeted to high-risk women [51]. As with previous research [46–48], preferences for MPTs in our study diminished under circumstances where side-effects (including menstrual changes) were greater with a dual-protection than a single indication product, and with formats that could not be stopped/removed if dual-protection was no longer needed or desired, highlighting trade-offs of choosing an MPT product. However, dual-protection products are unlikely to have more side effects than two separate single-indication products administered together.

There was a preference amongst our study participants for non-palpable products. This preference was most pronounced among young women in our study, who were more likely than the other age groups to report having a regular (non-spouse) sexual partner or one or more casual partners, though it was a significant driver of product preferences across all age groups. It is worth noting, however, that contraceptive implants in the market are typically palpable, and non-palpability makes them difficult to remove [52]. Other research has found that implant flexibility, discreetness, and biodegradability may increase acceptability of the implant among target end-users [53]. Previous studies also found that healthcare providers in South Africa similarly favored biodegradable product options to reduce the need for removal visits. However, they strongly preferred palpable options to aid in removal (if required) [54], and to verify that the implant has not moved [55], suggesting potential tensions in creating a product with high appeal amongst end-users as well as healthcare providers. While the evidence to date suggests that other product features (including length of protection) are more important to end-users, palpability and visibility may be modifiable characteristics of the long-acting PrEP implants and injectables currently under development. Having an implant that is flexible (versus stiff), of a smaller size, or biodegradable may assuage some user concerns around comfort or privacy, while future research could explore the trade-offs between features such as reduced palpability and increasing the number of injections required for a full dose.

Finally, our research found that while product preferences are driven by format, duration of protection, dual-protection, and discreetness, PrEP preferences varied by age group and potentially across the life course. Product preferences are likely to evolve as individual life circumstances—underlying HIV risk, relationship status, desire for pregnancy—change. Making a range of products available, and through a variety of service delivery channels, including family planning providers, community outreach models, and self-administered options, would address key barriers to PrEP uptake and the high rates of HIV acquisition among young women and girls in east and southern Africa.

### Limitations

This study was subject to several important limitations. First, the study sample was recruited from major urban centers and was not, therefore, representative of each country as a whole. Previous research from South Africa found differences in the strength of preferences for some features of long-acting PrEP products between urban and rural respondents [26]. As this was an online survey, our respondents tended to be wealthier and better educated than the general population, and our findings may not be generalizable to lower wealth quintiles, those with less education, or to women and girls in rural settings. Though using a self-directed, online survey format may have minimized this, our survey asked potentially sensitive questions about sexual activity and HIV risk, and these questions may have been subject to social desirability bias. Further, while remote support was available to participants, the self-directed nature of the online survey meant that users may have experienced challenges with comprehension, attention/cognitive fatigue, or technological issues that could have impacted on data quality. Further, beyond asking participants to confirm their identities and having personal survey links, there was no way to fully ensure that the individual completing the online survey was the same person who was originally sampled via the market research database. Additionally, we did not collect refusal rates during recruitment from the databases. We told potential participants during the screening and informed consent process that we wanted to recruit people for the study who were sexually active and either HIV-negative or did not know their status. However, for privacy/confidentiality reasons we did not collect HIV status as a part of this study, and it is possible that some of our respondents were knowingly living with HIV. Finally, because this was a stated preferences study, it is possible that the preferences described by participants would be different than their real-world behavior, should a PrEP implant become available.

Despite these limitations, this study provides important insights into women and girls’ preferences for long-acting PrEP products. We sampled more than 1200 women in three African countries with a high incidence of HIV, and, through a quantitative survey and a DCE, assessed the stated preferences of the respondents related to the next generation of biomedical HIV prevention interventions.

## Conclusion

This study found a strong preference for highly effective longer-acting HIV PrEP products, as well as those that provided dual-protection against either other STIs or pregnancy, among adolescent girls and women in 3 countries in east and southern Africa. Respondents also stated a high willingness to use an implantable PrEP product were one available in their context, though most would want to access it outside of specialty HIV/STI care clinics. Offering a choice of PrEP products, including dual-protection options, and through different service delivery models, such as family planning providers, would address existing barriers to PrEP among women and girls. These insights will inform ongoing product R&D, but will also be relevant to policymakers, implementing organizations, and donors for future long-acting PrEP roll-out and scale-up in these settings to ensure these products are delivered in the way that appeals to and meets the unique needs of women and girls.

## Data Availability

The quantitative data, codebooks, and survey instruments used in this study are available from the corresponding author on reasonable request.
